# Bariatric surgery and T2DM improvement mechanisms: a mathematical model

**DOI:** 10.1186/1742-4682-9-16

**Published:** 2012-05-15

**Authors:** Puntip Toghaw, Alice Matone, Yongwimon Lenbury, Andrea De GAETANO

**Affiliations:** 1 Department of Mathematics, Faculty of Science, Kasetsart University, Bangkok, Thailand; 2 Laboratorio di BioMatematica, Istituto di Analisi dei Sistemi e Informatica “A. Ruberti”, Consiglio Nazionale delle Ricerche, Roma, Italy; 3 Centre of Excellence in Mathematics, CHE, Si Ayutthaya RD., Bangkok, 10400, Thailand; 4 Department of Mathematics, Faculty of Science, Mahidol University, Bangkok, Thailand

**Keywords:** Bariatric Surgery, Diabetes, Mathematical Model, Incretins

## Abstract

**Background:**

Consensus exists that several bariatric surgery procedures produce a rapid improvement of glucose homeostasis in obese diabetic patients, improvement apparently uncorrelated with the degree of eventual weight loss after surgery. Several hypotheses have been suggested to account for these results: among these, the anti-incretin, the ghrelin and the lower-intestinal dumping hypotheses have been discussed in the literature. Since no clear-cut experimental results are so far available to confirm or disprove any of these hypotheses, in the present work a mathematical model of the glucose-insulin-incretin system has been built, capable of expressing these three postulated mechanisms. The model has been populated with critically evaluated parameter values from the literature, and simulations under the three scenarios have been compared.

**Results:**

The modeling results seem to indicate that the suppression of ghrelin release is unlikely to determine major changes in short-term glucose control. The possible existence of an anti-incretin hormone would be supported if an experimental increase of GIP concentrations were evident post-surgery. Given that, on the contrary, collected evidence suggests that GIP concentrations decrease post-surgery, the lower-intestinal dumping hypothesis would seem to describe the mechanism most likely to produce the observed normalization of Type 2 Diabetes Mellitus (T2DM) after bariatric surgery.

**Conclusions:**

The proposed model can help discriminate among competing hypotheses in a context where definitive data are not available and mechanisms are still not clear.

## Background

Severe obesity is one of the major problems of modern society, being related with a wide spectrum of diseases (e.g. cardiovascular disease, metabolic syndrome, type 2 diabetes, certain kind of tumors [1-3] and increased mortality. This problem has been expanding in recent years, quadrupling from 1968 to 2000, reaching now almost 5% of the adult population. At present the most effective and long-lasting solution for clinically severe obesity is bariatric surgery, which produces weight loss between 50% and 75% of excess body weight. Compared with other methods in which weight gain often recurs, with bariatric surgery the objective is typically maintained [4].

One of the main diseases linked to obesity is Type 2 Diabetes Mellitus (T2DM). The term “diabesity” [5] has in fact been introduced to refer to obesity accompanied by T2DM. As a consequence, it is not rare that subjects undergoing bariatric surgery are affected by diabetes. In such cases a very interesting side-effect of surgery has been observed since the ‘70s, that is, T2DM remission. This effect is already apparent few days after surgery, i.e. much earlier than the beginning of weight loss.

The improvement of glycemia in post-bariatric-surgery patients has been linked with an early improvement of insulin resistance post-surgery [6,7]. On the other hand, improvement in insulin secretion has also been proposed [8]. Further, it is not really clear whether the improvement in insulin resistance is immediate [6] or delayed by a few months [9], and whether it could as well be obtained by a very strict dietary regimen [10]. There have been in the last decade a consistent number of publications on the topic.

A study by Muscelli et al. showed insulin sensitivity amelioration proportional to weight loss after restrictive procedures, while complete reversion of insulin sensitivity long before body weight normalization was observed with malabsorptive surgery [11]. In 2006, Guidone et al. published a study on 10 patients, in which diabetes completely disappeared one week after surgery and insulin sensitivity was normalized [12]. Possible mechanisms implicated in this phenomenon, such as incretins [13] or ghrelin [14] have been discussed. Normalization of insulin sensitivity after malabsorptive bariatric surgery could be linked with the reduction of the effect of some intestinal factors due to intestinal bypass [15]. Diabetes remission after bariatric surgery can be key factor in the development of diabetes treatment strategies, but the underlying physiology, at present, is incompletely known [16,17].

Therefore, mechanisms underlying insulin resistance remission are still not clear: several hypotheses have been proposed but none of them has been confirmed yet.

There are several types of bariatric surgery procedures, grouped in three main classes: restrictive bariatric surgery, malabsorptive procedures and a combination of the two. Restrictive bariatric surgery consists in reducing the stomach size, thus increasing satiety and reducing food intake. The most common such procedure is laparoscopic adjustable gastric banding (GB). Malabsorptive procedures are based on bypassing a portion of the gut, thus consistently reducing the absorption of nutrients. Biliopancreatic diversion (BPD) is the classical example of malapsorptive procedure. However, the most common type of bariatric surgery is Roux-en-Y gastric bariatric procedure (RYGB), a combination restrictive and malabsorptive technique. In this kind of surgery the stomach is reduced to a small proximal pouch, which is then anastomosed to the jejunum, while the rest of the stomach and the duodenum are bypassed, and reconnected to the jejunum allowing the excretion of gastrointestinal and pancreatic juices.

In order to explain the mechanisms whereby gastric bypass procedures are effective in normalizing glycemia, it has been supposed that the gut removal itself may have a main role in diabetes remission, also in light of the fact that important hormones are secreted there. In 2009 Cummings reviewed the hypotheses that have been considered so far to explain the mechanisms underlying diabetes remission [18]. According to this Author, the main hypotheses are the ghrelin hypothesis, the upper intestinal hypothesis and the lower intestinal hypothesis.

The ghrelin hypothesis [18] maintains that ghrelin regulation might be disturbed following RYGB. Ghrelin is a hormone secreted by the stomach and proximal small bowel especially before meals, whose main physiological effects are increased appetite and fat mass increase [19]. In support of the ghrelin hypothesis, several studies have shown that ghrelin levels after RYGB are very low. Diminished ghrelin secretion can decrease appetite and food intake, and its compromised secretion might have a role in increasing glucose tolerance, as ghrelin can stimulate counter-regulatory hormones [20].

The lower intestinal hypothesis claims that intestinal shortcuts, created by bariatric surgery, expedite delivery of ingested nutrients and increase Glucagon-like peptide-1 (GLP-1) release. GLP-1 is an incretin, a peptide secreted from enteroendocrine L-cells, which are found throughout the small intestine and in high density in the ileum. GLP-1 increases insulin secretion and it has also been shown to increase proliferation and decrease apoptosis of beta-cells [21]. Both RYGB and BPD create gastro-intestinal shortcuts and it has been shown that postprandial GLP-1 secretion is increased post-surgery [22,23]. It thus seems reasonable that after surgery GLP-1 secretion may be enhanced, thus leading to enhanced insulin secretion. This mechanism could perhaps also explain the increase in β-cell mass that is thought to accompany post-RYGB hyperinsulinemic hypoglycemia [24].

The upper intestinal hypothesis maintains that avoiding the contact of nutrients with the duodenum is somehow the key process through which diabetes is improved. The suggestion at the basis of this hypothesis is that some kind of unknown factors or processes from the duodenum would influence glucose homeostasis [18]. The first support to this hypothesis came from Rubino and Marescaux [25], who experimented a variant of RYGB creating the intestinal bypass but leaving the stomach intact, thus inducing the same digestive discontinuity without reanastomosis. This surgery, called duodenal–jejunal bypass (DJB), was tested in several studies that showed an improvement in T2DM with no reduction in body weight [20,26-30]. These studies suggest that the exclusion of the proximal intestine *per se* has a role in diabetes remission.

In the present work, we introduce a mathematical model, which approximately describes the dynamics of the glucose-insulin-incretins system, allowing for the reproduction of the known and putative effects of bariatric surgery on insulin secretion. The three hypotheses advanced by Cummings [18] correspond to three specific scenarios obtainable by assigning suitable values to the model parameters. In this way it is possible to theoretically investigate the effects of the hypothesized mechanisms and verify whether they are compatible, at least qualitatively, with the known physiology in this class of patients.

## Materials and methods

### Physiological meaning of the State Variables

#### Glucose in Stomach, Duodenum , Ileum and Plasma (S, D, L, G)

Once ingested, glucose goes into the stomach, where digestion begins, and then reaches the small bowel, passing through the *pylorus.* The small intestine is divided in duodenum, jejunum and ileum, which may also be divided into subsections [31]. In the proposed model we consider a simplified division in duodenum and ileum. Each section is composed of different types of cells, which secrete different peptides in response to the passage of nutrients, and glucose is absorbed into plasma from each section, with different absorption rates. In our model the amount of glucose present in each section is considered as a state variable, in order to simulate the effect of secreted peptides and of the absence of a gut portion after surgery.

#### Plasma Insulin (I)

Insulin is a hormone secreted from pancreatic beta-cells in response to rising levels of plasma glucose concentrations. Insulin’s main function is to stimulate peripheral-tissue glucose uptake and inhibit liver glucose production. When insulin function is compromised, either depending on a defect in the action of insulin on tissues, or on a defect of insulin production itself, glucose is insufficiently absorbed by tissues or is excessively produced by the liver.

#### Incretins: GLP-1 (W) and GIP (U)

Glucagon-like peptide 1 (GLP-1) is an incretin, it stimulates insulin biosynthesis and insulin secretion in a glucose-dependent manner. The enteroendocrine L-cells of the distal ileum and colon synthesize and secrete GLP-1 in response to nutrient ingestion. There might be endocrine and neural signals accounting for the rapid increase of plasma GLP-1 after a meal, which happens before digested food has transited through the gut and has been in proximity with the L-cells. GLP-1 is synthesized as an inactive molecule of 37 amino acids; the six N-terminal ones are then cleaved yielding the active form. GLP-1 plasma concentrations are low in the fasting state, they increase 5 to 15 minutes after the meal. The circulation half-life for GLP-1 is only 1–2 minutes, since it is rapidly degraded by the enzyme Dipeptidyl-peptidase IV (DPP4, see below). Once in the bloodstream, GLP-1 reaches its target cells, which are pancreatic alfa and beta-cells, but also cells from other tissues (the nervous system, heart, kidney, lung, gastrointestinal tract) [32]. Insulin release is highly correlated with the secretion of GLP-1, which is one of the strongest known insulin stimulating factors [33].

Glucose Insulinotropic Polypeptide (GIP) is another incretin, secreted from K-cells, which are found in highest density in the duodenum and proximal jejunum, but have actually been found in the whole small bowel mucosa [33]. Glucose and fat absorption are the main factors stimulating secretion of GIP, which is produced as an active 42 aminoacid peptide. Similarly to GLP-1, plasma concentrations increase 5 to 15 minutes after the meal, and the polypeptide is then cleaved by DPP4. GIP circulation half-life is 5–7 minutes. When GIP is released from the gut into the bloodstream, it reaches its specific receptors on pancreatic beta-cells. Some GIP receptors are also found on the adipose, bone and brain tissues. In the beta-cell, GIP induces an increase in cAMP concentration, which causes an elevation in calcium, thus triggering the release of insulin granules [32,33].

The action of GLP-1 and GIP has been named the “incretin effect” [34]: it refers to the post-meal increase in insulin secretion due to these gut-secreted hormones. In healthy subjects this effect accounts for 50-70% of the overall insulin response [34]. In T2DM patients the “incretin effect” is reduced and this may depend on a defect in GLP-1 and GIP secretion [35].

#### DPP4 (P)

Dipeptidyl-peptidase IV (DPP4) is a ubiquitous serine protease which rapidly degrades GIP and GLP-1 as well as many other peptides. Its role in the inactivation of bioactive peptides was recognized due to its unique ability to liberate Xaa–Pro or Xaa–Ala dipeptides from the N-terminus of regulatory peptides. DPP4 has several functions and is strongly expressed on the surface of cells of different kinds of tissues: gastrointestinal tract, exocrine pancreas, kidneys, biliary tract, lymphoid organs, various glands. It is also found in body fluids such as blood plasma. DPP4 can inactivate many mammalian regulatory peptides, such as neuropeptides, circulating hormones and chemokines. Some important DPP4 substrates are neuropeptide Y, endomorphin, peptide YY, growth hormone-releasing hormone, GLP-1 and −2, and GIP [36].

#### Anti-incretin (A)

The upper intestinal hypothesis implies the presence of some kind of unknown “factor” which is compromised after the exclusion of the duodenum from the GI tract. This factor would be lowering or antagonizing the effect of incretins, so that the exclusion of the duodenum and the consequent impairment of the anti-incretin would lead to an increase in insulin secretion [37]. In order to simulate this hypothesis we included in the model a variable for the “anti-incretin” plasma concentration, assuming that the “anti-incretin” is secreted from the duodenum and inhibits the release of incretins.

#### Ghrelin (H)

Ghrelin is a 28 amino acid hormone secreted by the stomach and proximal small bowel. Its main physiological effects are orexigenia (increased appetite) and fat mass increase. Ghrelin is a strong stimulator of growth hormone (GH) release, being the natural ligand of the GH secretagogue receptor. Nevertheless, it has been shown that ghrelin has several different activities (stimulation of lactotroph and corticotroph secretion, cardiovascular actions, antiproliferative effect on thyroid and breast tumors, gastric motility and acid secretion regulation through vagal mediation) [19]. Plasma ghrelin concentration increases progressively before a meal, during which it varies by two- to threefold, reaching a minimum about one hour after the meal: this suggests that it may have a role in sensing low blood glucose. Moreover, it has been shown that ghrelin is produced (at a low rate) from the pancreas, which may indicate some relation with insulin release. Taken together, these findings lead to the involvement of ghrelin in glucose homeostasis and in diabetes development. In recent years the role of ghrelin has been widely investigated and, even if the mechanisms of action are still not completely clear, progress has been made [38]. A number of studies in vitro and in vivo show that ghrelin induces hyperglycemia and reduces insulin secretion, but some results are controversial and it is not clear whether the decrement in insulin production is a consequence of a direct effect of ghrelin on pancreatic beta-cells. A recent human study in vivo by Tong et al. [39] shows that exogenous ghrelin has an inhibitory effect on glucose-stimulated insulin release and glucose disappearance.

### The model

**Figure 1:**
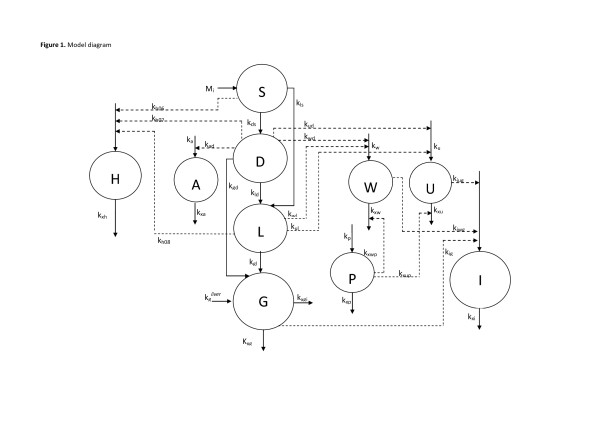
**Model block diagram.** State variables are represented with circles, solid arrows represent mass transfers, while dashed arrows indicate stimulations. The model is here schematically represented: the path of ingested glucose (M_i_) from the stomach (S) through duodenum (D) and ileum (L), and absorption in the plasmatic compartment (G) is along the central set of compartments (bottom-down). The insulin compartment (I) is on the bottom right of the figure, while the incretins (W and U) and DPP4 (P) are represented in between glucose compartments and insulin. Finally, on the left side, anti-incretin (A) and ghrelin (H) are represented. All the compartments are linked with dashed arrows, indicating stimulation of the entry rates or of the elimination rates, showing the relationship between state variables.

The proposed model is composed of 10 ordinary differential equations: the physiological meaning of each variable has been described above and in Figure 1 a block diagram representing the model is shown.

(1) $$\begin{array}{cc} \frac{dS(t)}{dt}=-k_{\mathrm{ds}}S(t)-k_{\mathrm{ls}}S(t)+\sum_{i=1}^{Nmeals}M_{i}\delta (t-t_{i}), & S\left(T_{\min}\right)=S_{\mathrm{Tmin}}{}_{} \end{array}$$

(2) $$\begin{array}{ccc} \frac{dD(t)}{dt}=k_{\mathrm{ds}}S(t)-k_{\mathrm{ld}}D(t)-k_{\mathrm{gd}}D(t), & & D\left(T_{\min}\right)=D_{\mathrm{Tmin}}{}_{} \end{array}$$

(3) $$\begin{array}{ccc} \frac{dL(t)}{dt}=k_{\text{ld}}\text{D}(t)+k_{\text{ls}}S(t)-k_{\text{gl}}L(t), & & L\left(T_{\min}\right)=0 \end{array}$$

(4) $$\begin{array}{cc} \frac{dG(t)}{dt}=-{\text{k}}_{\text{xg}}\text{G(t})-{\text{k}}_{\text{xgi}}\text{I(t)G(t})+\text{f}\;\frac{{\text{k}}_{\text{gd}}\text{D(t})+{\text{k}}_{\text{gl}}\text{L(t})}{{\text{V}}_{\text{g}}}+{\text{k}}_{\text{g}}^{\text{liver}}, & G\left(T_{\min}\right)=G_{\mathrm{Tmin}}{}_{} \end{array}$$

(5) $$\begin{array}{cc} \frac{dI(t)}{dt}=(k_{\mathrm{ig}}G(t)+k_{\mathrm{iwg}}G(t)W(t)e^{-\lambda_{01\text{a}}A(t)}+k_{\mathrm{iug}}G(t)U(t)e^{-\lambda_{02\text{a}}A(t)})e^{-\lambda_{03h}H(t)}-k_{\mathrm{xi}}I(t), & I\left(T_{\min}\right)=I_{\mathrm{Tmin}} \end{array}$$

(6) $$\begin{array}{ccc} \frac{dW(t)}{dt}=k_{\mathrm{wd}}D(t)e^{-\lambda_{04\text{a}}A(t)}+k_{\mathrm{wl}}L(t)-k_{\mathrm{xwp}}P(t)W(t)-k_{\mathrm{xw}}W(t)+k_{w}, & & W\left(T_{\min}\right)=W_{\mathrm{Tmin}} \end{array}$$

(7) $$\frac{dU(t)}{dt}=k_{\mathrm{ud}}D(t)e^{-\lambda_{05\text{a}}A(t)}+k_{\mathrm{ul}}L(t)-k_{\mathrm{xup}}P(t)U(t)-k_{\mathrm{xu}}U(t)+k_{u},\begin{array}{c} U\left(T_{\min}\right)=U_{\mathrm{Tmin}} \end{array}$$

(8) $$\frac{dP(t)}{dt}=k_{p}-k_{\mathrm{xp}}P(t),P\left(T_{\min}\right)=P_{\mathrm{Tmin}}$$

(9) $$\frac{dA(t)}{dt}=k_{\mathrm{ad}}D(t)-k_{\mathrm{xa}}A(t)+k_{a},\begin{array}{ccc} & & A\left(T_{\min}\right)=A_{\mathrm{Tmin}} \end{array}$$

(10) $$\frac{dH(t)}{dt}=(k_{h06}e^{-\lambda_{06\text{s}}S(t)}+k_{h07}e^{-\lambda_{07\text{d}}D(t)}+k_{h08}e^{-\lambda_{08\text{l}}L(t)})e^{-\lambda_{09\text{i}}I(t)}-k_{\mathrm{xh}}H(t),\begin{array}{cc} & H\left(T_{\min}\right)=H_{\mathrm{Tmin}} \end{array}$$

Equation 1 describes the dynamics of the amount of ingested glucose in the stomach, the first term represents the transfer from the stomach to the duodenum, the second the transfer from the stomach to the ileum, which happens in case of duodenum removal. The last term is a summation representing the entry of glucose with a Dirac delta, three times a day, corresponding to three meals.

Equation 2 describes the dynamics of the amount of glucose in the duodenum. The first term represents the entry from the stomach, the second is the exit to the ileum, and the last term is the absorption to the plasmatic glucose compartment.

Equation 3 describes the variation of the amount of glucose in the ileum. The entry terms, represented by the first two terms, are from the duodenum or from the stomach in case of bypass surgery, respectively. The last term accounts for the absorption into the plasmatic compartment.

Plasma glucose concentration dynamics is described in equation 4: the first two terms represent the insulin-independent and insulin-dependent glucose tissue uptake, respectively. The third term is plasma glucose entry. This is written as proportional to a fraction (not all of the glucose in the small bowel is absorbed) of the amount of glucose either in the duodenum or the ileum. The term is divided by the glucose distribution volume.

Equation 5 represents plasma insulin concentration. All the entry terms are collected in parentheses: the first term accounts for glucose dependent insulin secretion. The second and third terms depend on glucose as well, but also on GLP-1 and GIP concentration, respectively. GLP-1 and GIP action is opposed by anti-incretin with an exponentially decreasing dynamics. All insulin entry terms decrease exponentially with ghrelin concentration. The last term accounts for linear insulin elimination. Although in the literature the dependency of incretin-dependent insulin secretion from plasma glucose concentration is not always considered [40], there is indeed evidence supporting this mechanism. As from Verspohl 2009 p.117-118 “…activation of GIP and GLP-1 receptors on B-cells leads to rapid increases in levels of cAMP (Fig. 5 in Verspohl 2009) which is glucose-dependent (Drucker et al., 1987) and which is linked to both acute and long-term effects” [41,42]. Recent publications also report this effect, for example, in the paper from Gautier et al. 2008, it is stated: “GIP exerts glucose-dependent stimulatory effects on insulin secretion in animals and humans” and “GLP-1 stimulates glucose-induced insulin secretion in isolated islets of Langerhans” [43]. The same effect is reported from Holst [44,45].

The next equations describe the dynamics of those factors, mentioned above, which can influence glucose and insulin homeostasis. Equation 6 describes the variation of plasma GLP-1 concentration. The first term accounts for the entry due to the passage of glucose into the duodenum, which is exponentially controlled by anti-incretin concentrations. The second term represents the entry due to glucose in the ileum. The third is the elimination term representing DPP4 action, the fourth accounts for the natural GLP-1 disappearance. The last term accounts for GLP-1 constant secretion.

Equation 7 is similar to equation 6, and describes GIP dynamics.

In equation 8 DPP4 dynamics is represented. This has been supposed to be simple, that is, a constant production and a linear elimination. DPP4 is an ubiquitous enzyme involved in many pathways, thus it would be very complex and somewhat arbitrary to describe a more detailed control mechanism for it.

The anti-incretin equation (Eq. 9) is composed of the entry due to glucose in the duodenum, a linear elimination term, and a constant production rate.

The last equation (Eq. 10) describes ghrelin concentration variation. The first three terms, in parentheses, represent the constant entry terms which are exponentially inhibited by the presence of glucose in the stomach, in the duodenum and in the ileum, respectively. All entry terms are also exponentially decreased by insulin concentration. The last term accounts for linear ghrelin elimination.

### Parameter values

Model parameters, which are not determined from steady state assumptions or other constraints, have been given as reasonable a value as possible, based on the literature. Simulations start at time T_min_ = 0 minutes, at which we assume an early morning fasting state, therefore there is no glucose in the stomach, duodenum or ileum. Therefore, at time T_min_ we have S_Tmin_ = D_Tmin_ = L_Tmin_ = 0 mmol. Meal times have been set to t_1_ = 30 min, t_2_ = 300 min, t_3_ = 540 min, corresponding to breakfast, lunch and dinner, respectively. The amount of ingested glucose at each meal is, respectively, M_1_ = 400 mmol, M_2_ = 500 mmol, M_3_ = 600 mmol. In this work we will show simulations for only one meal (*e.g.* lunch).

Since bariatric surgery is performed on obese subjects, we considered, where possible, parameters values corresponding to this category of patients.

Fasting plasma glucose was reported to be 8.05 ± 1.82 mM for T2DM obese subjects [46]. American Diabetes Association guidelines define the diabetes threshold at 7 mM fasting plasma glucose [47], while impaired fasting glucose is defined above 6.1 mM. In the present work we choose to represent a borderline diabetic obese subject with G_Tmin_ = 7 mM.

In obese subjects, fasting plasma insulin was found to be 171 ± 74 pM, corresponding to subjects with 8.05 ± 1.82 mM glycemia [46]. Here we consider I_Tmin_ = 100 pM.

Fasting plasma GLP-1 levels were reported at 8.7 ± 2.8 pM for obese subjects [48], we set W_Tmin_ =8 pM. From the same source fasting plasma GIP levels were 21.5 ± 5.8 pM for normal and obese subjects [48,49], we set therefore U_Tmin_ =20 pM.

Mean DPP4 activity in human serum was found to be 29.7 ± 6.6 U/l as collected from 481 healthy adult volunteers (ages 19–61 years) [50], so we set P_Tmin_ = 30 U/l. The maximum level of GLP-1 plasma concentration, after glucose ingestion, in obese patients, after Roux-en-Y gastric bypass surgery is W_max_ = 100 pM [46]. For what concerns GIP maximal concentration, we set U_max_ = 300 pM, as GIP secretion is greatly increased (10 to 20-fold) in response to meal ingestion, so we consider 15 times the basal plasma concentration [33]. P_max_ is taken arbitrarily as 10 times P_b_ that is P_max_ = 300 U/l.

D_max_ and L_max_ are the maximal glucose content in the duodenum and in the ileum, respectively, during the course of normal food intake. We set them to 2000 mmol, corresponding to 360 grams of glucose.

Glucose distribution volume is approximately 0.2 l/kg body weight [51]. Hypothesizing for moderate obese subjects an average weight of 90 kg, we set V_G_ = 18 liters.

Stomach emptying rate is represented from the parameter k_ds_ which we set at 0.02 min^-1^, as found in the literature [52] and k_ls_ was set to the same value. Similarly, the transfer rates from the duodenum to the ileum k_ld_, from the ileum to plasma k_gl_, and from duodenum to plasma k_gd_, were set to 0.02 min^-1^.

Silber et al, [53] report a glucose clearance rate (C_L_) = 0.089 l/min, with k_xg_ = Cl/V_G_ is approximately equal to 0.0049 min^-1^. Insulin-dependent glucose elimination rate, k_xgi_, has been set to 0.2 × 10^-4^ as from [54], again consistent with a degree of insulin resistance typically exhibited by obese prediabetics. The fraction of absorbed glucose was set to f = 0.9.

The parameter k_gliver_ was determined by setting the derivative of equation (4) equal to zero (steady state):

(11) $$0=-{\text{k}}_{\text{xg}}{\text{G}}_{\text{Tmin}}-{\text{ k}}_{\text{xgi}}{\text{I}}_{\text{Tmin}}{\text{G}}_{\text{Tmin}}+{\text{k}}_{\text{gliver}}\text{,}$$

thus

(12) $${\text{k}}_{\text{gliver}}={\text{k}}_{\text{xg}}{\text{G}}_{\text{Tmin}}+{\text{k}}_{\text{xgi}}{\text{I}}_{\text{Tmin}}{\text{G}}_{\text{Tmin}}=0.077\text{pM}/\text{min}$$

The disappearance rate constant for insulin has been set to k_xi_ = 0.04 min^-1^[55].

The combined action of GLP-1 and GIP is estimated to account for approximately 50%–70% of the total insulin secretory response depending on the size of the glucose load ([44,56-58]). GLP-1 was reported to be three to five times more potent than GIP ([33,56,59]).

Therefore, k_ig_, k_iwg_ and k_iug_ were determined by setting the derivative of equation (5) equal to zero (steady state)

(13) $$0=(k_{\mathrm{ig}}G_{T\min}+k_{\mathrm{iwg}}G_{T\min}W_{T\min}e^{-\lambda_{01\text{a}}A_{T\min}}+k_{\mathrm{iug}}G_{T\min}U_{T\min}e^{-\lambda_{02\text{a}}A_{T\min}})e^{-\lambda_{03h}H_{T\min}}-k_{\mathrm{xi}}I_{T\min}$$

and from the stated hypotheses

(14) $$k_{\mathrm{ig}}G_{T\min}=0.3\;\left\{\;k_{\mathrm{ig}}G_{T\min}+k_{\mathrm{iwg}}G_{T\min}W_{T\min}e^{-\lambda_{01\text{a}}A_{T\min}}+k_{\mathrm{iug}}G_{T\min}U_{T\min}e^{-\lambda_{02\text{a}}A_{T\min}}\right\}$$

Solving equations we have

(15) $$k_{\mathrm{ig}}=\frac{3}{7}\;\left\{\;k_{\mathrm{iwg}}W_{T\min}e^{-\lambda_{01\text{a}}A_{T\min}}+k_{\mathrm{iug}}U_{T\min}e^{-\lambda_{02\text{a}}A_{T\min}}\right\}$$

(16) $$k_{\mathrm{iwg}}=\frac{0.56k_{\mathrm{xi}}I_{T\min}}{G_{T\min}W_{T\min}e^{-\left(\lambda_{01\text{a}}A_{T\min}+\lambda_{03\text{h}}H_{T\min}\right)}}$$

(17) $$k_{\mathrm{iug}}=\frac{0.25k_{\mathrm{iwg}}W_{T\min}e^{-\lambda_{01a}A_{T\min}}}{U_{T\min}e^{{}^{-\lambda_{02a}A_{T\min}}}}$$

These parameter values change according to the different scenario set to simulate the three hypothesis.

The incretin effect has been shown to be reduced in patients with type 2 diabetes, although the mechanisms are still incompletely understood [43].

**Figure 2:**
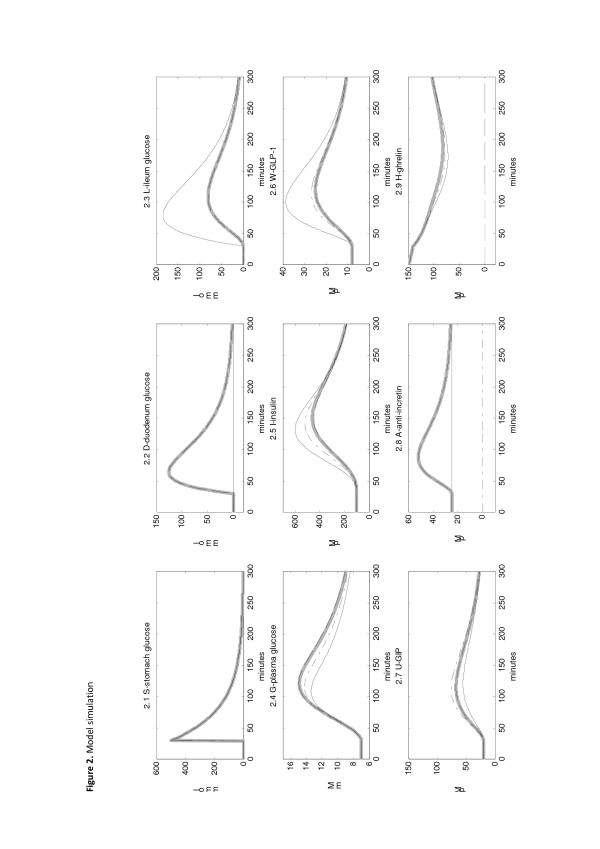
**Model simulation.** Thick gray line: pre-surgery case; solid line: lower-intestinal hypothesis (LIH); dash-dot line: anti-incretin hypothesis (AIH); dashed line: ghrelin hypothesis (GH). 2.1 Stomach glucose content is unchanged in all the hypotheses. 2.2 Glucose content in the duodenum is zero in the LIH, while is unchanged in the other scenarios. 2.3 Ileum glucose content is higher and the peak is earlier, compared to the pre-surgery scenario, for the LIH. In the other hypotheses the dynamics is the same. 2.4 Plasma glucose concentration is lower in the AIH and the LIH (more markedly in the latter). The curve in the GH is unchanged compared to the pre-surgery case. 2.5 Plasma insulin concentration is higher in the AIH and the LIH (more markedly in the latter). GH is unchanged compared to the pre-surgery case. 2.6 GLP-1 concentration is markedly higher in the LIH, while is slightly higher in the AIH and unchanged in the GH. 2.7 GIP concentration increases in the AIH, decreases in the LIH, is unchanged in the GH. 2.8 Anti-incretin concentration is constant at the basal value in the LIH, zero in the AIH, unchanged in the GH 2.9 Ghrelin concentration dynamics is slightly lower in the LIH and the AIH, while for the GH ghrelin concentration is zero.

The parameter lambda_01a_ represents the decay rate of the GLP-1 effect of insulin production, due to the anti-incretin effect. Since insulin secretion in response to GLP-1 in diabetic patients has been shown to be similar to that in normal subjects [5], we set A_501_ (anti-incretin concentration at which GLP-1 effect is half of its maximum value) to 150 pM, consequently lambda_01a_ = log(2)/A_501_ = 0.002 pM^-1^.

lambda_02a_ is the decay rate of GIP stimulated insulin production due to the increase in anti-incretin concentration. Since insulin secretion in response to GIP administration has been found to be 54 % lower in diabetic patients [43], we set A_502_ (anti-incretin concentration at which GIP effect is half of its maximum value) to 75 pM and lambda_02a_ = log(2)/A_502_ = 0.004 pM^-1^.

lambda_03h_ represents the decay rate of insulin production due to the increase in ghrelin concentration. In Tassone et *al.* (Figure 2 page 548 [60]) insulin levels reductions after ghrelin administration in obese humans are reported (ghrelin 1 μg/kg). Taking H_50_ = 1225 pM we obtain lambda_03h_ = log(2)/H_50_ = 2.46 x 10^-4^ pM^-1^.

GLP-1 is secreted from intestinal endocrine L-cells, which can be found throughout the human small intestine with highest density in the distal ileum. Mortensen et al. [61] showed that the percentage of GLP-1 immunoreactive cells increases along the length of the gastrointestinal tract. From Figure 2 in Mortensen’s paper [61], the density of GLP-1 immunoreactive cells is about 15 % of the total amount of endocrine cells in the distal duodenum or proximal jejunum, and about 45% in the whole ileum. This approximate ratio is also consistent with median and maximum GLP-1 intestinal wall concentrations reported in the same paper [61].

We therefore assume that k_wd_ , the release rate of GLP-1 per mmol of ingested glucose appearing in the duodenum and the jejunum, is approximately 1/3 of the release rate of GLP-1 per mmol of ingested glucose appearing in the ileum: k_wd_ = k_wl_/3.

We hypothesize that, in a long simulation of GLP-1 dynamics, with a very large amount of glucose both in the duodenum and in the ileum (say, 2 moles or 360 grams), a steady state would be reached, so that GLP-1 production would equal GLP-1 elimination:

(18) $${\text{k}}_{\text{wd}}2000+{\text{k}}_{\text{wl}}2000={\text{K}}_{\text{xwp}}{\text{P}}_{\text{max}}{\text{W}}_{\text{max}}$$

In the light of the above proportion between duodeno-jejunal and ileal contributions to GLP-1 secretion we can write

(19) $$\left(1/3\right){\text{k}}_{\text{wl}}2000+{\text{k}}_{\text{wl}}2000={\text{k}}_{\text{xwp}}{\text{P}}_{\text{max}}{\text{W}}_{\text{max}}\text{,}$$

where P_max_ = 300 U/l is the maximal concentration of DPP4 and W_max_ = 100 pM the maximal concentration of GLP-1, and k_xwp_ = 9.04x10^-4^ min^-1^/pM the GLP-1 elimination rate. The ileal incretin secretion would thus be k_wl_ = 0.0102 pM/min/mmol, and the corresponding duodeno-jejunal GLP-1 secretion k_wd_ = 0.0034 pM/min/mmol. These are indeed very rough estimates. However, model behavior is affected very little by variations of these secretory rates from 20% to 300% of these presumptive amounts.

The parameter lambda_04a_ [pM^-1^] is the decay rate of GLP-1 production due to the anti-incretin. Since the secretion of GLP-1 in diabetic patients is impaired, we set A_502_ = 75 pM, we thus have lambda_04a_ = log(2)/A_502_ = 0.00602 pM^-1^.

Plasma half-life of GLP-1 is around 2.3 minutes (from table 2 in Meier’s paper [62]). Since GLP-1 is mainly degraded by the enzyme DPP4 (reviewed from Deacon [63]), we assume that k_xwp_, the disappearance rate constant for GLP-1 due to DPP4, is approximately 90% of the disappearance rate constant for GLP-1. This hypothetical value is computed as k_xwp_ P_max_ = 0.9log(2)/2.3, and k_xwp_ = 9.04x10^-4^ min^-1^/pM . While k_xw_, the disappearance rate constant for GLP-1, is k_xw_ = 0.1log(2)/2.3 = 0.0301 min^-1^.

k_w_ was determined by setting the derivative of equation (6) equal to zero (steady state), thus k_w_ = 0.4581pM/min.

GIP is secreted from intestinal K-cells, which can be found throughout human small intestine. The highest percentage of GIP-immunoreactive cells is however in the upper jejunum [61]. From Figure 2 in Mortensen’s paper [61], the density of GIP immunoreactive cells is about 20 % of the total amount of endocrine cells in the distal duodenum or proximal jejunum, and about 10 % in the whole ileum. This approximate ratio is also consistent with median and maximum GLP-1 intestinal wall concentrations, reported in the same paper [61].

We therefore assume that k_ud_, the release rate of GIP per mmol of glucose appearing in the duodenum and the jejunum, is approximately twice the rate of release of GIP per mmol of glucose appearing in the ileum. The value was computed as k_ud_ = 2k_ul_.

As it was computed for GLP-1, we set at 2 moles the maximum plausible glucose amount in the duodenum and in the ileum, and we hypothesize to reach a steady state where

(20) $${\text{k}}_{\text{ud}}2000+{\text{k}}_{\text{ul}}2000={\text{k}}_{\text{xup}}{\text{P}}_{\text{max}}{\text{U}}_{\text{max}}$$

or, from the above proportion between duodeno-jejunal and ileal contributions to GIP secretion,

(21) $${2\text{k}}_{\text{ul}}2000+{\text{k}}_{\text{ul}}2000={\text{k}}_{\text{xup}}{\text{P}}_{\text{max}}{\text{U}}_{\text{max}}\text{,}$$

where P_max_ = 300 U/l, Umax = 500 pM, and k_xup_ = 4.16x10^-4^ min^-1^/pM. So we have k_ul_ = 0.0104 pM/min/mmol and k_ud_ = 0.0208 pM/min/mmol.

The parameter lambda_05a_ [pM^-1^ is the decay rate of GIP production as anti-incretin concentrations increase. Since it has been shown that GIP measurements in diabetic subjects are the same compared to patients with no T2DM [5] we take A_501_ = 150 pM, obtaining lambda_05a_ = log(2)/A_501_ = 0.0046 pM^-1^.

Plasma half-life of GIP is around 5 minutes, from table 2 in Meier’s paper [62]. Similarly to GLP-1, GIP is mainly degraded by the enzyme DPP4 (reviewed from Deacon [63]). We therefore assume that k_xup_, the disappearance rate constant for GIP due to DPP4, is approximately 90% of the disappearance rate constant for GIP. A plausible value for k_xup_ can therefore be computed from k_xup_P_max_ = 0.9log(2)/5, so that k_xup_ =4.16x10^-4^ min^-1^/pM. The parameter k_xu_, the disappearance rate constant for GIP, was computed as k_xu_ =0.1log(2)/5 = 0.0139 min^-1^.

The parameter k_u_ was determined by setting the derivative of equation (6) equal to zero (steady state), thus obtaining k_u_ = 0.5268pM/min.

The enzyme DPP4 is responsible for the rapid degradation of the incretin hormones. From Figure 1 in Mentlein’s paper [36], DPP4 is found on the plasma membrane of cells at numerous sites, including the kidney, brush-border membranes of the intestine, and the liver. It is also located on the endothelial cells of blood vessels and is found in a soluble form in plasma. Since the factors regulating the expression and activity of DPP4 in glucose metabolism are barely known, and since the action of DPP4 is ubiquitous and its role affects many different substrates, we assume that DPP4 levels are approximately constant.

The parameter k_p_, the appearance rate constant for DPP4, is determined by setting the derivative of equation (8) equal to zero (steady state) and k_xp_, the disappearance rate constant for DPP4, is taken arbitrarily as 0.05 min^-1^:

(22) $${\text{k}}_{\text{p}}={\text{k}}_{\text{xp}}{\text{P}}_{\text{Tmin}}=1.5\text{ U}/\text{L}/\text{min.}$$

We arbitrarily set the unit of measurement of the unknown anti-incretin hormone to pM. We suppose that fasting and maximum plasma Anti-incretin level are the same as GIP: A_Tmin_ = 25 pM and A_max_ = 500 pM. We assume the disappearance rate constant the anti-incretin, k_xa_ = 0.05 min^-1^, as DPP4. k_ad_ and the appearance rate constant due to glucose in the duodenum is computed, by taking analogy with DPP4, as ${\text{k}}_{\text{ad}}{\text{D}}_{\text{max}}={\text{k}}_{\text{xa}}{\text{A}}_{\text{max}},{\text{ from which k}}_{\text{ad}}=0.0125\text{ pM}/\text{min}/\text{mmol}$.

Fasting plasma ghrelin has been found to be around 148 ± 30 pM for obese subjects [64], so we set H_Tmin_ = 150 pM. From the same reference H_max_ =300 pM, and the approximate minimum level of plasma ghrelin collected from obese patients after gastric bypass surgery H_min_ = 29.6 pM [64].

Plasma ghrelin concentrations rise gradually in the fasted state and decrease immediately after a meal (review by Hameed et al. [65]). Plasma ghrelin concentrations decrease after oral and intravenous administration of glucose [66]. However, the percentage of ghrelin decrease was found to be inversely correlated with the percentage of increase in insulin and glucose [67]. The tissue content of immunoreactive ghrelin in the rat was found to be around 90 % in the stomach, 7 % in the duodenum-jejunum and 2 % in the ileum [68]. Plasma ghrelin levels from subjects who underwent gastric bypass decreased to around 1/5 the pre-surgery levels [64], suggesting that around two thirds of circulating human ghrelin come from the stomach and one third comes from the small intestine.

The parameters k_h06_, k_h07_ and k_h08_, the appearance rate constants for ghrelin due to glucose in the stomach, duodenum and ileum, respectively, were determined by setting the derivative of equation (10) equal to zero (steady state):

(23) $$\;0=(k_{h06}+k_{h07}+k_{h08})e^{-\lambda_{09i}I_{t\min}}-k_{\mathrm{xh}}H_{t\min}$$

(24) $$k_{h06}+k_{h07}+k_{h08}=k_{\mathrm{xh}}H_{t\min}e^{\lambda_{09i}I_{t\min}}$$

(25) $$k_{h06}=(2/3)k_{\mathrm{xh}}H_{t\min}e^{\lambda_{09i}I_{t\min}}$$

(26) $$k_{h07}=(2/9)k_{\mathrm{xh}}H_{t\min}e^{\lambda_{09i}I_{t\min}}$$

and

(27) $$k_{h08}=(1/9)k_{\mathrm{xh}}H_{t\min}e^{\lambda_{09i}I_{t\min}}$$

where k_xh_ is the disappearance rate constant for ghrelin, arbitrarily taken as 0.02 min^-1^. These parameters change according to the scenario.

We have no direct information on the decay rate of ghrelin production with increasing glucose in the stomach, the duodenum or ileum. Since plasma ghrelin concentrations decrease immediately after a meal and considering the 24-hour plasma ghrelin profiles from Figure 1 in Cumming’s paper [64], S_50_, D_50_ and L_50_ were taken as 50, 100 and 150 mmol, respectively, in order to match the reported plasma ghrelin profiles.

The parameter lambda_06s_ [mmol^-1^] is the decay rate of ghrelin production as amounts of glucose in the stomach grow larger. Taking S_50_ = 50 mmol we have lambda_06s_ = log(2)/S_50_ = 0.006 mmol^-1^.

The parameter lambda_07d_ [mmol^-1^] is the decay rate of ghrelin production as amounts of glucose in the duodenum grow larger. Taking D_50_ = 100 mmol we have lambda_07d_ = log(2)/D_50_ = 0.003 mmol^-1^.

The parameter lambda_08l_ [mmol^-1^] is the decay rate of ghrelin production as amount of glucose in the ileum grow larger. Taking L_50_ = 150 mmol we have lambda_08l_ = log(2)/L_50_ = 0.002 mmol^-1^.

The parameter lambda_09i_ [pM^-1^] is the decay rate of ghrelin production as plasma insulin concentrations increase. Taking I_50_ = 10 pM we have lambda_09i_ = log(2)/I50 = 0.03 pM^-1.^

### Three scenarios

The present model was implemented in Matlab2010b® and four simulations were compared. One represents the pre-surgery scenario, in which no parameters are changed in the model: meals are administered and all parameters are fixed to the values shown above.

In order to simulate the three hypotheses, the parameters that were thought to differ from the pre-surgery situation were changed on the basis of the hypothesized physiological mechanism. In Table 1 all parameter values that were changed to test the three hypotheses are shown.

**Table 1:** Parameters which change in the three scenarios; parameters values pre-surgery and for each hypothesis are show

	** Pre-surgery**	**Post-surgery**			** Definition **
		**Hypothesis**			
**Parameters**		**Lower-intestinal (GLP-1)**	**Upper-intestinal (Anti-incretin)**	**Ghrelin**	
N_meals_	1	1	1	1	meal n.1 is simulated
M_1_	500	500	500	500	amount of glucose in the meal
A_Tmin_	25	25	0	25	plasmatic anti-incretin basal level
A_max_	500	500	0	500	maximum amount of anti-incretin
H_Tmin_	150	150	150	0	plasmatic ghrelin basal level
H_max_	300	300	300	0	maximum amount of ghrelin
H_min_	29.6	29.6	29.6	0	minimum amount of ghrelin
k_ds_	0.02	0	0.02	0.02	transfer rate from stomach to duodenum
k_ls_	0	0.02	0	0	transfer rate from stomach to ileum
k_ld_	0.02	0	0.02	0.02	transfer rate from duodenum to ileum
k_gd_	0.02	0	0.02	0.02	transfer rate from duodenum to plasma glucose

It must be remarked that the simulations conducted in this way reflect “pure” or isolated effects of each of the three hypothesized mechanisms.

### The ghrelin hypothesis

This hypothesis is based on the observation that plasma ghrelin levels decrease to about one third of the pre-surgery concentration in subjects who undergo a proximal Roux-en-Y gastric bypass [64]. Since Ghrelin is shown to suppress insulin secretion [67] this could be the mechanism at the basis of diabetes remission.

To test this hypothesis with the model, ghrelin was excluded from the model, without in any way affecting glucose transit in the gut compared to pre-surgery. To exclude ghrelin we set the following parameters to 0: plasmatic ghrelin basal level, H_Tmin_ = 0, maximum amount of ghrelin, H_max_ = 0, and minimum amount of ghrelin, H_min_ = 0.

### The lower intestinal hypothesis

It is hypothesized that GLP-1 secretion is increased after bariatric operations, which create intestinal shortcuts and expedite delivery of ingested nutrients to the lower bowel.

For the test model, the duodenum has been excluded from contact with nutrients: glucose goes directly from the stomach to the ileum.

To simulate this mechanism, the transfer rates from the stomach to the duodenum, from the duodenum to the ileum and from the duodenum to plasma glucose, were set to 0: k_ds_ = 0, k_ld_ = 0, k_gd_ = 0. The transfer rate from stomach to ileum was set to a value different from 0, compared to pre-surgery: k_ls_ = 0.02.

### The upper intestinal hypothesis

Under this hypothesis it is claimed that the exclusion of a short segment of proximal small intestine (primarily the duodenum) from contact with ingested nutrients exerts direct anti-diabetic effects, by means of the reduction in some unknown anti-incretin molecule secreted by the duodenum itself.

To test this hypothesis the Anti-incretin was excluded from the model, without affecting glucose transit in the gut.

The following parameters have been changed, compared to pre-surgery: the plasmatic anti-incretin basal level, A_Tmin_ = 0 and the maximum amount of anti-incretin, A_max_ =0.

## Results

### Simulations

The model has been run setting first the parameters to the “default” pre-surgery situation, and then to the three scenarios representing the three hypotheses. The corresponding state variable plots are shown in Figure 2. The model has been formalized in order to simulate three meals in one day but only the first meal is shown here for clarity. In Figure 2 the 4 scenarios are shown together (thick gray line for the pre-surgery case, solid line for the lower-intestinal hypothesis, dash-dot line for the anti-incretin hypothesis, dashed line for the ghrelin hypothesis,). The DPP4 plot has not been shown because the concentration is constant, as explained in the Model section.

In the lower-intestinal hypothesis (solid line) glucose is transferred directly to the ileum, so glucose content in the duodenum is zero, while glucose content in the ileum is comparable to the glucose content in the duodenum pre-surgery curve. For the other two hypotheses the curves remain the same as the pre-surgery case.

For plasma glucose concentration dynamics the peak is lower and elimination is faster for the anti-incretin hypothesis and even more so for the lower-intestinal hypothesis. Symmetrically, plasma insulin concentration is higher and with a quicker dynamics in the anti-incretin hypothesis and even more so for the lower-intestinal hypothesis. GLP-1 concentration is slightly higher in the anti-incretin scenario, while it is remarkably elevated in the lower-intestinal hypothesis, compared to the pre-surgery situation. GIP concentration is higher than pre-surgery for the anti-incretin scenario and lower than pre-surgery for the lower-intestinal scenario. Anti-incretin concentration is 0 for the anti-incretin hypothesis, as it was set from the parameters, while in the lower-intestinal hypothesis concentration is constant. Ghrelin concentration is zero for the ghrelin scenario, while it does not change appreciably among the remaining three scenarios. It must be noticed that for every state variable, excepting ghrelin concentration itself, the pre-surgery and ghrelin scenarios produce essentially undistinguishable curves.

## Discussion

In 1995 Poires et al. first reported that the diabetes resolution effect observed after RYGB was remarkable (82.9%) [69,70]. Subsequently there have been several studies reporting that amelioration in insulin resistance was observed before a consistent weight loss occurred. A pilot study reported in Chiellini et al. 2009 showed that BPD on T2DM patients with BMI <35 kg/m^2^ achieved reduced glycaemia and an increase in insulin sensitivity one month post-surgery [71]. Observations from a study conducted by Hickey et al. showed an improvement in glucose and insulin levels few days after gastric bypass surgery, before the establishment of a significant weight loss [6]. Other studies have shown that weight loss after bariatric surgery cannot account for diabetes recovery [72,73]. The independency of diabetes remission from weight loss has therefore been recognized since the 90’s but the mechanisms by which this happens are still under investigation [25,74-76].

Controversial results have in fact been reported on this topic, with some Authors maintaining that diabetes improvement is not independent from weight loss. In the study from Campos et al., comparing a group of patients undergoing RYGB with a group of non operated subjects [9], it was reported that peripheral glucose uptake improvement is observed only after significant weight loss. Another trial, comparing DJB- and Sham-operated rodents, suggested that DJB alone is not sufficient to improve insulin sensitivity independently from weight loss [77].

Nevertheless, a very recent publication [17], specifically focused on this topic, strongly supports the efficacy of bariatric surgery in diabetes amelioration. An unblinded randomized controlled trial was performed on 60 patients undergoing either gastric bypass, biliopancreatic diversion or conventional therapy. Study results show T2DM remission in 75% and 95% of the gastric bypass and biliopancreatic diversion groups, respectively. None of the patients treated with conventional medical therapy showed T2DM remission. To support the hypothesis of weight-independent diabetes remission, no correlation was found between normalization of fasting glucose levels and weight loss in the bariatric surgery groups, and remission (judged from complete withdrawal of medication) took place within 15 days of surgery.

In the light of the results supporting weight-loss-independent diabetes remission, some hypotheses have been made, such as the ghrelin hypothesis, the anti-incretin, or “foregut” hypothesis, and the lower intestinal, or “hindgut” hypothesis [18]. In the present paper we propose a mathematical model, which can simulate these three hypotheses to illustrate what kind of results could be expected from quantitative analysis of the consequences of the corresponding assumptions.

From the plots in Figure 2 it is evident that the ghrelin curve, in all plots, is essentially no different from the pre-surgery curve. This suggests that, based on the model simulation, the hypothesis that ghrelin is the responsible factor for the remission of T2DM post-surgery is not convincing. Findings about ghrelin levels reported in the literature are in fact inconsistent [76], and there is some evidence that ghrelin levels are unchanged after bariatric sugery [78].

The anti-incretin and the lower intestinal hypothesis simulations both give higher plasma insulin concentration peaks as well as lower glycemic levels, although the effect seems to be more evident in the lower intestinal case.

It must be underscored that the results of a simulation study, such as the present one, are dependent on a number of assumptions, both in the simplification of the model structure and in the assessment of the model parameters. For this reason it would be inappropriate to draw conclusions from modest quantitative differences in predictions, and the somewhat greater effect on glycemia levels, produced in the lower-intestinal scenario, should not be viewed as supporting this hypothesis over the anti-incretin one.

On the other hand, if we consider predictions of gut hormone levels, GLP-1 and GIP, we notice that, while in the anti-incretin hypothesis both hormone levels increase, in the lower intestinal hypothesis GLP-1 concentration increases, while GIP concentration decreases, compared to pre-surgery levels. This last prediction is not a small quantitative change but a rather clear-cut qualitative difference in behavior, which, according to the model, follows from the physiology modifications introduced by bariatric surgery under the two different sets of hypotheses. From literature sources, diverse results are reported about GIP level changes after bariatric surgery. Several studies report decreased levels of GIP post-surgery [23,79,80]. Other studies report essentially no change in GIP post-surgery [46,81]. Bose [5] explicitly states that there is no support for increased fasting GIP levels after bariatric procedures. Laferrere et al. [82] report transient increase in GIP levels after GBP. Therefore, while the preponderance of evidence would seem to favor a decrease of GIP levels post-surgery, thereby providing support for the lower-intestinal hypothesis, this conclusion does not seem to be definitively established.

The present work presents several limitations, for example the values of the variables at t = T_min_ do not change post surgery, while there might be a variation in some of these values caused by physical rearrangements. One such issue (regarding insulin basal value) is discussed below. Another aspect to consider is that a meal is usually composed of proteins and fats, not only of glucose, and these would also be involved in driving several mechanism, for example, incretin secretion. Lastly, surgery is a process which indeed temporarily affects patient homeostasis, due to prolonged fasting and bed rest.

Some of the limitations of the model could be improved with the collection of data, among which glucose and insulin concentrations after a glucose test, as well as incretin levels. This would go a long way towards a more precise and reliable assessment of the several model parameters.

Another aspect which is worth mentioning, regards insulin levels post-surgery. In fact, in the simulations, insulin concentrations increase under both upper and lower intestinal hypotheses. From the literature, however, there is some evidence for insulin decrease after BPD during the Oral Glucose Tolerance Test (OGTT). From [46], insulin concentration and peak insulin during OGTT increase while fasting insulin decreases around 25% after RYGB. From Briatore 2008, fasting insulin decreases around 50%, acute insulin response or early insulin secretion increase, after BPD [83]. From Valverde 2005 fasting insulin decreases 75%, insulin concentrations decrease but the incremental area in plasma insulin concentration over the first 30 min of the OGTT test was higher after BPD [84]. From Bose 2009, a review of the effects of bariatric surgery, fasting insulin decreases, while insulin sensitivity increases. From the available evidence, fasting insulin decreases by 25-70% 1–6 months after surgery: this is consistent with a lower insulin secretion determined by higher insulin sensitivity [5]. Under these conditions, insulin concentrations will in general decrease over the natural history of the patient after surgery. For the present simulations, however, fasting insulin is regarded as a constant, independently of the hypothesized maneuver, thus reflecting the physiology immediately before and immediately after surgery. Under these assumptions, I_tmin_ is the same both before and after surgery; under both upper and lower intestinal hypotheses the levels of incretin increase; the insulin secretory response to glucose increases; and insulin concentrations increase. If we set I_tmin_ post-surgery to around 50 % its pre-surgery level then insulin concentrations will not appear to increase overall.

It would be interesting to consider the relevance of the phenomenon of “Transfer of Addiction”, which has been observed post-bariatric surgery: compulsive overeating can in fact be replaced with other compulsive disorders, such gambling, drugs, alcoholism, compulsive shopping and exercise. This phenomenon has been thought to be connected with the Reward Deficiency Syndrome (RDS), which is related to dopamine receptor defects, and has been described as a genetic disease [85]. It has been thought that, in those subjects where “Transfer of Addiction” takes place after bariatric surgery, obesity and thus compulsive eating behavior could be a sort of defense mechanism towards other addictions [86]. It is known that insulin can affect dopamine receptors and vice versa [87], the activity of the dopamine transporter can in fact be increased by high insulin levels {{96}}. These findings are relevant in the frame of bariatric surgery outcomes and should be taken into consideration in a future development of the present work.

## Conclusion

The mathematical modeling approach can be a useful tool for a better understanding of the interplay of the several mechanisms operating in the glucose-insulin-incretin system. Model conclusions, based upon a set of reasonable but necessarily incomplete assumptions on parameter values, point to the measurement of post-surgery changes in GIP levels as one discriminating factor differentiating expected behavior under the lower intestinal and anti-incretin hypotheses. The weight of evidence accumulated so far would seem to support the lower intestinal hypothesis over the anti-incretin hypothesis, while ghrelin effect appears irrelevant for the rapid normalization of diabetes after bariatric surgery.

## Abbreviations

BPD, Biliopancreatic Diversion; DJB, Duodenal–Jejunal Bypass; DPP4, Dipeptidyl-peptidase IV; GB, Gastric Banding; GH, Growth Hormone; GI, Gastrointestinal; GIP, Glucose Insulinotropic Polypeptide; GLP-1, Glucagon-Like Peptide 1; RYGB, Roux-en-Y gastric bariatric procedure; T2DM, Type 2 Diabetes Mellitus.

## Competing interests

The authors declare that they have no competing interests.

## Authors’ contributions

PT investigated the form of the several components of the model from a mathematical point of view, participated in the assessment of parameter values from the literature and wrote portions of a first draft of the manuscript; AM was responsible for checking physiological mechanisms and parameter assessment and for compilation of the manuscript; YL reviewed the overall mathematical correctness of the aggregated equations; ADG planned the investigation, designed the general form of the model, verified its overall physiological plausibility and reviewed the manuscript. All authors read and approved the final manuscript.
